# Institutional Organisations in America

**Published:** 1914-07-11

**Authors:** 


### INSTITUTIONAL ORGANISATION IN AMERICA.

### I. Some Reforms in Children's Out-Patient Clinics.

Hospital organisation is one of the matters in which the Americans have made, and are making, such vast strides that it behoves the English institutional world to note very carefully the new and progressive ideas which are constantly being ventilated there. For it is only by a determination to keep up to date that one can prevent oneself from retrogressing; keeping up to date implies a sharp watch on other peoples' inventions and discoveries, and their adoption whenever they are proved to be more advantageous than older methods. It is perhaps true that in the United States there are great numbers of institutions which fall far behind the high standard set by the select few; the need for some levelling up is more urgent than with us. But even allowing for this, it is clear that the leaven of the most efficient of the American institutions will permeate the whole mass eventually, and that only a resolute intention to raise our own standard also will enable us to keep up in the race. Without going so far as the editors of *American Medicine*, who have recently declared that "the hospital situation in New York is admittedly so bad that nothing short of a radical reorganisation will remedy its defects," and without approving of all the heroic measures which are suggested for improving matters, it may yet be stated that the American institutional world is much exercised as to an all-round improvement, and that some very sane and practical steps have already been taken to secure it.

One of these striking instances of American ingenuity in institutional matters that has come under our notice is in connection with standards for out-patient clinics. The different sections of the New York Associated Out-patient Clinics have worked out a code of standards by which to classify the clinics of that city. The work of the pediatric (children's) section is particularly interesting. A list of stringent requirements must all be satisfied by any institution which aspires to admission to Class A. For Class B the conditions are almost as exacting, but not quite. All other institutions which do not satisfy at least the requirements of Class B are lumped together as unsatisfactory, and recommended for prompt closure unless they reform their administration.

### Class A.

The standards for the first class are as follows:—

1. Efficient medical treatment at all seasons of the year, which can only be possible (*a*) if no more than six patients, old and new, per medical officer per hour are admitted, and (*b*) if arrangements are made for the presence of a medical director * or his first assistant during every clinic hour throughout the entire year.

2. A nurse in attendance during all clinic hours, and a visiting nurse attached exclusively to the clinic. When possible and practicable the same nurse should combine the two functions.

3. At least two examining rooms and an additional room for teaching where teaching is done at the clinic.

4. An isolation room for cases of contagious disease.

5. A paid assistant present during all clinic hours, one of whose duties should be the segregation and isolation of contagious cases.

6. Proper laboratory facilities for the immediate examination of specimens of blood and urine at the clinic, or at least in the dispensary or hospital building.

7. Proper equipment should consist of: a proper examining table; proper scales for infants and children, weighing up to 100 lbs.; measuring instruments, such as callipers, rulers, and so forth; special lighting arrangements for nose, ear, throat, and head work; proper and plentiful supply of linen, towels and sheets, and similar necessaries; proper desks and chairs; proper ventilation, plumbing, and light, proper surgical instruments; walls and floors easily washed; filing cabinets for history cards; "reference blanks" for patients referred to other departments of the institution or to other institutions; facilities for *x*-ray work; printed diet lists and printed hygienic instructions for children over one year.

8. Special provision for treating whooping-cough cases (either a special room or a special attendance hour) until such time as the Department of Health provides adequate facilities for the treatment of this class of patients.

9. An infant milk station at the clinic, or close co-operation with a Department of Health milk station, arranged in such a way as to be mutually agreeable to the clinic and to the Department of Health.

10. Special provision for vaginitis cases, if they are not taken care of in some other department of the institution.

### Class B.

The requirements for the second class are much the same, but there are certain omissions from the Class A schedule. Thus No. 2 of the Class A requirements is left out altogether; in other words, the clinic can be run without an out-patient nurse at all, provided it reaches the minimum standard in other respects. No. 5 of the list for Class A is also omitted from the Class B requirements. The list of equipment (No. 7) is the same for both classes, except that no *x*-ray outfit is required of the B clinics. Lastly, the milk-station clause (No. 9) does not appear on the schedule for Class B.

It will be observed that in most respects there is nothing in the least unusual about these standards; indeed, there are a good many other demands which might quite reasonably be included. Most British children's departments would pass with flying colours in the greater part of the schedule. The

* This American phrase may be taken as the equivalent of controlling surgeon or physician, a post to which in Britain there is no analogue first specification is that one in which most of our clinics would be found wanting—namely, the admission of no more than six patients, old and new, per doctor per hour. Yet the stipulation is not only not unreasonable; it is perfectly sound in principle, though it may not be found put into practice in many clinics in this country. The present writer retains a vivid recollection of his first day's duty as house surgeon in a children's hospital of world-wide fame, when he found himself faced with a collection of forty-four new cases and over eighty old ones to treat in the out-patient department between 9 A.M. and 1 P.M. That incident happened a good many years ago, and things may possibly be different now; but it will be observed that clause 1 was violated in both its subdivisions in that case. We repeat with regret that there are very, very few British children's out-patient departments which would satisfy either of these two requirements as contained in the first paragraph of the pediatric section's standards.

The other deficiency of most of our institutions is in respect of clause 9. Very few of our clinics have any milk station or any system of co-operation with outside organisations for supplying decent milk to the patients; and so most of them would fail to pass in this respect for Class A, though they would not thereby be excluded from Class B.

Whether this attempt at a hard-and-fast, mechanical classification of the degrees of efficiency of out-patient departments is of much real value opinions will necessarily differ. Obviously enough, the method has serious limitations. It takes no account of the quality of the medical work done, or of the fitness of the medical staff to do it. It omits a good many essentials and includes certain things lacking which it is easily possible to carry on a clinic of the very highest efficiency. It takes no account, necessarily, of the personal element, nor of the institutional spirit, without which no perfection in equipment will constitute a first-class clinic. Yet when all this is admitted, the fact remains that this standard does provide a means of comparison between different clinics to a certain extent. With a little amendment it could be easily adapted to suit British conditions, and would serve to show some of the less progressive clinics a measure of their imperfections. At all events, it is of interest as an indication of that restless desire for improvement which is so characteristic of the American and such a valuable aid to social progress and betterment.

